# ACOCMPMI: An Ant Colony Optimization Algorithm Based on Composite Multiscale Part Mutual Information for Detecting Epistatic Interactions

**DOI:** 10.1155/humu/7656300

**Published:** 2025-06-13

**Authors:** Yan Sun, Jing Wang, Yaxuan Zhang, Junliang Shang, Jin-Xing Liu

**Affiliations:** ^1^College of Engineering, Qufu Normal University, Rizhao, Shandong, China; ^2^School of Computer Science, Qufu Normal University, Rizhao, Shandong, China; ^3^School of Health and Life Science, University of Health and Rehabilitation Sciences, Qingdao, Shandong, China

**Keywords:** ant colony algorithm, Bayesian network, epistatic interaction, multiscale part mutual information

## Abstract

Epistatic interaction detection plays a pivotal role in understanding the genetic mechanisms underlying complex diseases. The effectiveness of epistatic interaction detection methods primarily depends on their interaction quantification measures and search strategies. In this study, a two-stage ant colony optimization algorithm based on composite multiscale part mutual information (ACOCMPMI) is proposed for detecting epistatic interactions. In the first stage, composite multiscale part mutual information is developed to quantify epistatic interactions, and an improved ant colony optimization algorithm incorporating filter and memory strategies is employed to search for potential epistatic interactions. In the second stage, an exhaustive search strategy and a Bayesian network score are adopted to further identify epistatic interactions within the candidate SNP set obtained in the first stage. ACOCMPMI is compared with five state-of-the-art methods, including epiACO, FDHE-IW, AntEpiSeeker, SIPSO, and MACOED, using simulation data generated from 11 epistatic interaction models. Furthermore, ACOCMPMI is applied to detect epistatic interactions in a real dataset of age-related macular degeneration. The experimental results show that ACOCMPMI is a promising method for epistatic interaction detection.

## 1. Introduction

In recent years, numerous single nucleotide polymorphisms (SNPs) associated with complex diseases have been successfully detected through genome-wide association studies (GWAS) [1]. However, the explanatory power of individual SNPs is limited in some complex diseases, such as cancer [2] and Alzheimer's disease [3]. Epistatic interactions, broadly defined as nonlinear interactions between SNPs, have emerged as a key mechanism to overcome these limitations. Therefore, the precise detection of epistatic interactions has become a focal point of research [4–6].

Epistatic interaction detection focuses on two key aspects: interaction quantification measures and search strategies. Interaction can be likened to a specific type of association, predominantly manifesting as nonlinear direct associations. Quantifying these interactions relies on various association measures. Traditional statistical measures, such as logistic regression [7, 8], chi-square statistic [9], distance covariance [10], and Pearson's correlation coefficient [11], are limited to quantifying nonlinear direct associations among target variables. Measures based on information entropy, which do not strictly depend on specific association forms, have gained significant attention in recent years. The mutual information (MI) and conditional mutual information (CMI) are commonly employed for quantifying nonlinear interactions among variables [12–15]. However, they may lead to overestimation and underestimation problems [16]. To precisely quantify nonlinear direct interactions, several measures have emerged, including maximum information coefficient (MIC) [17], conditional mutual inclusive information (CMI2) [18], part mutual information (PMI) [16], partial association (PA) [19] and multiscale part mutual information (MPMI) [20]. Notably, MPMI demonstrates higher accuracy compared with other measures and has not been applied to SNP data. Therefore, this study adopts MPMI and its variant to quantify interaction between SNPs.

The search strategy can be broadly categorized into three groups: exhaustive search, stochastic search, and heuristic search. Exhaustive search methods typically attempt to evaluate all possible SNP combinations within a dataset. However, the high dimensionality of GWAS data imposes a heavy computational burden on exhaustive methods [21]. Stochastic search methods are limited in the number of features they can handle [22]. Heuristic search transforms the epistatic interaction detection problem into an optimization problem. Heuristic search mainly focuses on metaheuristic optimization algorithms, such as the firefly algorithm [23], tree seed algorithm [24], tunicate swarm algorithm [25], side-blotched lizard algorithm [26], African vultures optimization algorithm (AVOA) [27], ant colony optimization (ACO) algorithm [28], symbiotic organisms search algorithm [29], spotted hyena optimizer algorithm [30], yellow saddle goatfish behavior optimization model [31], and grey wolf optimizer [32]. In this study, the ACO algorithm (ACO∗) is employed for searching epistatic interactions, and an improved version of the ACO∗ is presented. The ACO∗ has been widely used in this field [33] and is considered one of the most promising methods among these metaheuristic optimization algorithms.

The main contributions of this work are as follows.

• A composite version of MPMI, termed CMPMI, is proposed. CMPMI is specifically designed for detecting nonlinear direct interactions in SNP datasets.
• Memory and filtering strategies are integrated into the ACO∗ to improve the accuracy of epistatic interaction detection.
• Epistatic interactions are detected in a two-stage framework. In the first stage, an improved ACO∗ combined with CMPMI is used to generate a candidate SNP set. In the second stage, an exhaustive search strategy and a Bayesian network (BN) score are adopted to further identify epistatic interactions within the candidate set.

## 2. Related Works

Various methods have been proposed to detect epistatic interactions. For instance, multifactor dimensionality reduction (MDR) [34], backward genotype-trait association (BGTA) [35], Boolean operation-based screening and testing (BOOST) [8], factored spectrally transformed linear mixed models (FaST-LMM) [36], and tree-based epistasis association mapping (TEAM) [37] are epistatic interaction detection methods based on exhaustive search strategies. Bayesian epistasis association mapping (BEAM) [22] and epistatic module detection (EpiMODE) [38] employ stochastic search strategies. BEAM integrates the Bayesian partitioning model with Markov chain Monte Carlo to assess and identify disease-associated SNPs and epistatic interactions. EpiMODE utilizes a Bayesian marker partition model alongside a Gibbs sampling strategy to detect epistatic interactions. For heuristic search methods, CINOEDV is designed to detect and visualize epistatic interactions of various orders, leveraging the particle swarm optimization algorithm and co-information measure [39]. AntEpiSeeker uses a two-stage ACO∗ for identifying epistatic interactions in large datasets [40]. Similarly, MACOED is a multiobjective ACO supervised heuristic method for epistasis detection [41], and IACO applies an improved ACO∗ to search for epistatic interactions [42]. MTHSA-DHEI is proposed for detecting high-order epistatic interactions based on a multitasking harmony search algorithm [43]. Building on this framework, MTHS-EE-DHEI is introduced as an enhanced variant that incorporates explicit encoding into the multitasking harmony search algorithm to further optimize the epistasis detection [44].

## 3. Materials and Methods

### 3.1. MPMI

MPMI is an innovative measure designed to quantify direct associations between target variables [20]. Unlike traditional measures, it is not confined to specific interaction forms during quantification. Furthermore, its higher accuracy and superior statistical power render it a significant advancement in this field. The MPMI between *X* and *Y* given *Z* is defined as

(1) $$\begin{array}{c} \text{MPMI}\left(X;Y\left|Z\right.\right)=e^{2\text{MI}\left(X;Z\right)}\cdot e^{2\text{MI}\left(Y;Z\right)}\cdot \text{CMI}\left(X;Y\left|Z\right.\right)+D\left(p\left(x\left|z\right.\right)\left\|p^{*}\left(x\left|z\right.\right)\right.\right)+D\left(p\left(y\left|z\right.\right)\left\|p^{*}\left(y\left|z\right.\right)\right.\right), \end{array}$$

where both *X* and *Z* represent SNPs and *Y* represents the phenotype. *x* and *z* are genotypes of SNPs *X* and *Z*, and *y* is the class label of *Y*. MI(*X*; *Z*) is the MI between *X* and *Z*, and MI(*Y*; *Z*) is the MI between *Y* and *Z*. Both of them are defined as

(2) $$\begin{array}{c} \text{MI}\left(X;Z\right)=\underset{x,z}{\sum }p\left(x,z\right)\log\frac{p\left(x,z\right)}{p\left(x\right)p\left(z\right)}, \end{array}$$

(3) $$\begin{array}{c} \text{MI}\left(Y;Z\right)=\underset{\text{y},z}{\sum }p\left(y,z\right)\log\frac{p\left(y,z\right)}{p\left(y\right)p\left(z\right)}, \end{array}$$

where *p*(*x*, *z*) is the joint probability distribution of *x* and *z*, *p*(*y*, *z*) is the joint probability distribution of *y* and *z*, and CMI(*X*; *Y*|*Z*) is the CMI between *X* and *Y* given *Z*, which is defined as

(4) $$\begin{array}{c} \text{CMI}\left(X;Y\left|Z\right.\right)=\underset{x,y,z}{\sum }p\left(x,y,z\right)\log\frac{p\left(x,y,z\right)p\left(z\right)}{p\left(x,z\right)p\left(y,z\right)}, \end{array}$$

where *p*(*x*, *y*, *z*) is the joint probability distribution of *x*, *y*, and *z*.

In addition, both *D*(*p*(*x*|*z*)‖*p*^∗^(*x*|*z*)) and *D*(*p*(*y*|*z*)‖*p*^∗^(*y*|*z*)) are the extended Kullback–Leibler divergences [16]. They are defined as

(5) $$\begin{array}{c} D\left(p\left(x\left|z\right.\right)\left\|p^{*}\left(x\left|z\right.\right)\right.\right)=\underset{x,y,z}{\sum }p\left(x,y,z\right)\log\frac{p\left(x\left|z\right.\right)}{\sum_{y}p\left(x\left|z\right.,y\right)p\left(y\right)}, \end{array}$$

(6) $$\begin{array}{c} D\left(p\left(y\left|z\right.\right)\left\|p^{*}\left(y\left|z\right.\right)\right.\right)=\underset{x,y,z}{\sum }p\left(x,y,z\right)\log\frac{p\left(y\left|z\right.\right)}{\sum_{x}p\left(y\left|z,x\right.\right)p\left(x\right)}, \end{array}$$

where *p*(*x*|*z*) and *p*(*y*|*z*) are the probability distributions of *x* and *y* conditioned on *z*, respectively, and *p*(*x*|*z*, *y*) is the probability distribution of *x* conditioned on both *z* and *y*.

### 3.2. ACO∗

The ACO∗ is a classical swarm intelligence optimization algorithm designed to solve complex combinatorial optimization problems by simulating the cooperative behavior of ant colonies [45]. The basic idea of the ACO∗ is to map feasible solutions of optimization problems to paths traversed by ants. Ants tend to release more pheromones along shorter paths during their traversal. Meanwhile, pheromones guide ants in selecting subsequent paths. Ultimately, through positive feedback, all ants converge on the optimal path, which corresponds to the optimal solution of the optimization problem. The basic ACO∗ primarily involves two core strategies: path selection and pheromone update.

Ants navigate paths based on a combination of pheromones and heuristic information. Typically, the probability of an ant selecting the next position from a given current position during an iteration is defined as

(7) $$\begin{array}{c} P_{k}^{\text{ij}}\left(t\right)=\left\{\begin{array}{cc} \frac{\tau_{\text{ij}}{\left(t\right)}^{\alpha }\eta_{\text{ij}}^{\beta }}{\sum_{u\in M_{k}\left(t\right)}\tau_{\text{iu}}{\left(t\right)}^{\alpha }\eta_{\text{iu}}^{\beta }} & i\in M_{k}\left(t\right)\\ 0 & \text{otherwise} \end{array}\right., \end{array}$$

where *τ*_ij_(*t*) is the pheromone of path *i*⟶*j* in iteration *t*. Similarly, *η*_ij_ represents the heuristic information of path *i*⟶*j*. *α* and *β* are the weight coefficients of pheromone and heuristic information, respectively, both of which are usually set to 1. *M*_*k*_(*t*) represents the set of positions that are not detected by ant *k* in iteration *t*.

In iteration *t* + 1, the pheromone of path *i*⟶*j* is defined as

(8) $$\begin{array}{c} \tau_{\text{ij}}\left(t+1\right)=\left(1-\rho \right)\tau_{\text{ij}}\left(t\right)+\Delta \tau_{\text{ij}}\left(t\right), \end{array}$$

(9) $$\begin{array}{c} \Delta \tau_{\text{ij}}\left(t\right)=\overset{m}{\underset{k=1}{\sum }}\Delta \tau_{\text{ij}}^{k}\left(t\right), \end{array}$$

(10) $$\begin{array}{c} \Delta \tau_{\text{ij}}^{k}\left(t\right)=\left\{\begin{array}{cc} \frac{Q}{S_{k}\left(t\right)}, & \text{if ant }k\text{ passing path ij}\\ 0 & \text{otherwise} \end{array}\right., \end{array}$$

where *τ*_ij_(*t*) is the pheromone of path *i*⟶*j* in iteration *t*, *ρ* is an evaporation coefficient, *τ*_ij_(*t*) is the pheromone variation of path *i*⟶*j* in iteration *t*, *Q* is a user-defined constant, and *S*_*k*_(*t*) is the path length of ant *k* in iteration *t*.

### 3.3. BNs

A BN is a network structure based on a directed acyclic graph, used to represent dependencies among observed variables. In this network, nodes represent either SNPs or phenotypes, and edges connecting nodes signify causal dependencies. The K2 score, based on BN, is widely used to quantify causal dependencies between two variables.

The K2 score is derived from the Bayesian score. The Bayesian score computes the posterior probability *P*(*M*|*D*) of the BN model *M* given the data *D*, which can be written as

(11) $$\begin{array}{c} P\left(M\left|D\right.\right)=\frac{P\left(D\left|M\right.\right)P\left(M\right)}{P\left(D\right)}, \end{array}$$

where *P*(*D*|*M*) is the class-conditional density and *P*(*D*) and *P*(*M*) are the probabilities of the data *D* and the model *M*, respectively. Building upon prior studies [41, 46, 47], in the context of a case-control study, if all variables in the directed acyclic graph are discrete, we can derive

(12) $$\begin{array}{c} P\left(D\left|M\right.\right)=\overset{I}{\underset{i=1}{\prod }}\left(\frac{\Gamma \left(\sum_{j=1}^{J}\alpha_{ij}\right)}{\Gamma \left(r_{i}+\sum_{j=1}^{J}\alpha_{ij}\right)}\overset{J}{\underset{j=1}{\prod }}\frac{\Gamma \left(r_{ij}+\alpha_{ij}\right)}{\Gamma \left(\alpha_{ij}\right)}\right) \end{array}$$

where *I* is the number of combinations of SNP nodes with different genotypes, *r*_*i*_ is the case number of SNP nodes taking the *i*_th_ combination numbers, and *r*_ij_ is the number of cases with phenotypes taking the *j*_th_ state while its parents take the *i*_th_ combination. *J* is the state number of phenotypes. *α*_ij_ is the prior belief about case numbers with model nodes taking the *i*_*th*_ combination and *j*_th_ state, which is a hyperparameter when the model satisfies the Dirichlet distribution. If *α*_ij_ = 1, *P*(*M*) and *P*(*D*) are constants, then,

(13) $$\begin{array}{c} P\left(M\left|D\right.\right)\propto \overset{I}{\underset{i=1}{\prod }}\left(\frac{\left(J-1\right)!}{\left(r_{i}+J-1\right)!}\overset{J}{\underset{j=1}{\prod }}r_{ij}!\right). \end{array}$$

The Bayesian score can be transformed into the K2 score. Subsequently, the logarithmic form of the K2 score can be derived.

(14) $$\begin{array}{c} K2\text{ score}=\overset{I}{\underset{i=1}{\prod }}\left(\frac{\left(J-1\right)!}{\left(r_{i}+J-1\right)!}\overset{J}{\underset{j=1}{\prod }}r_{ij}!\right), \end{array}$$

(15) $$\begin{array}{c} K2\;{\text{score}}_{\log}=\overset{I}{\underset{i=1}{\sum }}\left(\overset{r_{i}+1}{\underset{b=1}{\sum }}\log\left(b\right)-\overset{J}{\underset{j=1}{\sum }}\overset{r_{ij}}{\underset{d=1}{\sum }}\log\left(d\right)\right). \end{array}$$

### 3.4. ACOCMPMI

**Figure 1:**
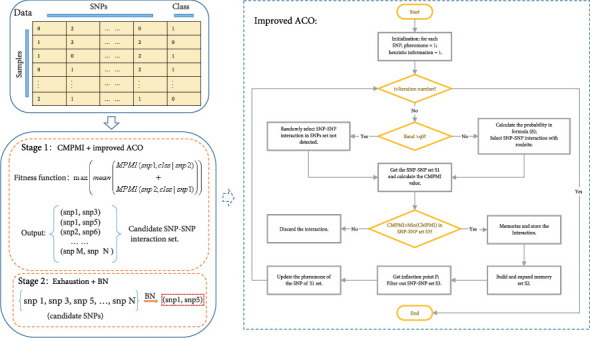
Flow chart of the ACOCMPMI.

Figure 1 is the flow chart of ACOCMPMI. It can be seen that the ACOCMPMI mainly consists of two parts: Stage 1 (CMPMI + improved ACO) and Stage 2 (exhaustion search + BN). Among them, Stage 1 is the highlight of ACOCMPMI.

### 3.5. CMPMI

The MPMI possesses several properties that prove beneficial for the investigation of epistatic interaction detection. For instance, (1) MPMI(*X*; *Y*|*Z*) ≥ 0; (2) MPMI(*X*; *Y*|*Z*) = 0 if and only if there is no direct association between *X* and *Y* under the condition of *Z*; (3) when both *X* and *Y* are independent of *Z*, MPMI(*X*; *Y*|*Z*) = CMI(*X*; *Y*|*Z*) = MI(*X*; *Y*); (4) MPMI(*X*; *Y*|*Z*) is a vsconstant when *X* is directly associated with *Y* regardless of the influence intensity of *Z* on their association; (5) for the target variables *X* and *Y*, MPMI(*X*; *Y*|*Z*) = MPMI(*Y*; *X*|*Z*).

It is seen that the MPMI can be regarded as “asymmetric” for the two-order SNP combination (*X*, *Z*) and the phenotype *Y*, which is inconsistent with the basic principle of association. Hence, to capture symmetric information in the detection of two-order epistatic interactions, we define the CMPMI as

(16) $$\begin{array}{c} \text{CMPMI}=\frac{\text{MPMI}\left(X;Y\left|Z\right.\right)+\text{MPMI}\left(Z;Y\left|X\right.\right)}{2}. \end{array}$$

CMPMI is essentially the mean form of MPMI between the involved target variables, indicating the integration of association information related to SNPs and phenotypes. Furthermore, CMPMI incorporates the interconnectedness of SNP combinations, making it symmetric in terms of describing associations.

### 3.6. An Improved ACO∗

Given that the basic ACO∗ exhibits low convergence speed and faces challenges with local minima problems [28, 48–50], we developed an improved ACO∗.

To avoid getting trapped in local optima, it is crucial to expand the search space for ants. Based on the original path selection strategy, incorporating suitable random strategies can guide ants out of cyclic paths, thereby providing them with a more diverse set of path selections [51]. The corresponding formulas for path selection can be written as

(17) $$\begin{array}{c} p_{k}^{i}\left(t\right)=\left\{\begin{array}{c} R\;q\leq q_{0}\\ S\;q>q_{0} \end{array}\right., \end{array}$$

(18) $$\begin{array}{c} S=\left\{\begin{array}{c} 1\;i=\mathrm{rand}\left(M_{k}\left(t\right)\right)\\ 0\;\text{otherwise} \end{array}\right., \end{array}$$

where *p*_*k*_^*i*^(*t*) is the probability that ant *k* selects SNP *i* in iteration *t*, *R* is the original path selection strategy, *q* is a randomly generated value satisfying a uniform distribution, and *q*_0_ is the user specified threshold that is set to the reciprocal of the number of iterations.

For pheromone updating, the original updating strategy is adopted. Thus, Δ*τ*_ij_^*k*^(*t*) can be written as

(19) $$\begin{array}{c} \Delta \tau_{i}^{k}\left(t\right)=\left\{\begin{array}{c} CMPMI\left(S\right)\;k\in {M^{*}}_{i}\left(t\right)\\ 0\;otherwise \end{array},\right. \end{array}$$

where Δ*τ*_*i*_^*k*^(*t*) is the pheromone variation of SNP *i* selected by ant *k* at iteration *t*, *M*^∗^_*i*_(*t*) represents the set of ants that select SNP *i* at iteration *t*, CMPMI(*S*) represents the CMPMI value of SNP combination *S*.

The memory-based strategy can retain superior solutions generated in each iteration, enhancing the overall convergence of the algorithm [51, 52]. Specifically, for each iteration, solutions captured by ants are sorted in descending order based on their CMPMI values. Subsequently, a turning point can be determined.

(20) $$\begin{array}{c} f=\arg\overset{m}{\underset{g=3}{\max}}\left(\begin{array}{c} \left(\text{CMPMI}\left(S_{g}\right)-\text{CMPMI}\left(S_{g-1}\right)\right)\\ -\left(\text{CMPMI}\left(S_{g-1}\right)-\text{CMPMI}\left(S_{g-2}\right)\right) \end{array}\right), \end{array}$$

where CMPMI(*S*_*g*_) is the CMPMI value of the SNP combination *S*_*g*_, *g* represents the ant. In each iteration, SNP combinations before the turning point are regarded as candidate solutions, and their corresponding fitness values are stored.

To further expedite convergence, a filtering operation based on the memory strategy is incorporated into ACOCMPMI. Within the candidate solution set obtained from each iteration, min(CMPMI) is utilized as the filter criterion. For subsequent iterations, those SNP combinations with CMPMI values greater than the filter criterion are retained and stored in the candidate solution set.

## 4. Results and Discussion

### 4.1. Evaluation Metrics

In the experiments, three evaluation metrics, including detection power, *F*-measure, and running time, are employed to assess the performance of compared methods.

Detection power is a widely used and effective metric for assessing the performance of methods for detecting epistatic interactions [39] and is defined as

(21) $$\begin{array}{c} \text{power}=\frac{D_{T}}{D}, \end{array}$$

where *D*_*T*_ is the number of datasets that epistatic interaction models in them are successfully detected and *D* is the total number of datasets. Besides, the *F*-measure is defined as

(22) $$\begin{array}{c} \text{Precision}=\frac{\text{TP}}{\text{TP}+\text{FP}}, \end{array}$$

(23) $$\begin{array}{c} \text{Recall}=\frac{\text{TP}}{\text{TP}+\text{FN}}, \end{array}$$

(24) $$\begin{array}{c} F‐\text{measure}=\frac{2\text{precision}\times \text{recall}}{\text{precision}+\text{recall}}=\frac{2\text{TP}}{2\text{TP}+\text{FP}+\text{FN}}, \end{array}$$

where true positives (TPs) represent that the detected SNP combinations are truly associated with the phenotype, false positives (FPs) represent that the detected SNP combinations are not associated with the phenotype, and false negatives (FNs) represent that the undetected SNP combinations are indeed associated with the phenotype.

### 4.2. Simulation Datasets

There are 11 epistatic interaction models to evaluate the performance of compared methods, where Models 1–8 are models displaying marginal effects (DMEs), and Models 9–11 are models displaying no marginal effects (DNMEs).  Table 1 lists details of these models, in which MAF represents minor allele frequency, AA is the homozygous common genotype, Aa is the heterozygous genotype, and aa is the homozygous minor genotype [49, 53]. Using these models, the simulator EpiSIM was applied to generate datasets of different scales [54]. For small-scale datasets, each model was used to generate 100 datasets, in which the sample number is 4000 and the SNP number is 100. For large-scale datasets, each model was used to generate 50 datasets, in which the sample number is 4000 and the SNP number is 1000.

### 4.3. Results on Simulation Datasets

**Figure 2:**
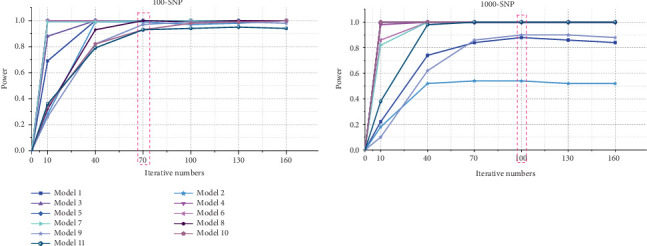
Detection power of ACOCMPMI with different iteration numbers.

For small-scale datasets, the ant number and the iteration number are set to 200 and 70, respectively, while for large-scale datasets, the ant number and the iteration number are set to 2000 and 100, respectively. The detection power of ACOCMPMI with different iteration numbers is precomputed for all models, and those iteration numbers close to the optimal convergence point are selected as the iteration parameters, as illustrated in Figure 2.

**Figure 3:**
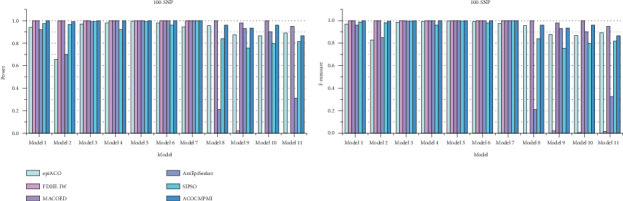
Detection power and *F*-measure of compared methods on small-scale datasets.

For small-scale datasets, detection power and *F*-measure of compared methods are presented in Figure 3. In terms of detection power, most methods perform well and detect almost all epistatic interactions in various datasets. Specifically, ACOCMPMI demonstrates high and stable detection power in DMEs, comparable to FDHE-IW and MACOED. Notably, FDHE-IW is a method specifically designed for detecting DMEs [55]. ACOCMPMI exhibits lower detection power than MACOED in small-scale DNME datasets, contrasting with its superior performance in large-scale datasets. Although AntEpiSeeker performs effectively in Model 4–7 datasets, it fails to detect epistatic interactions in Model 2, 8, and 11 datasets, implying that AntEpiSeeker may be inconsistent and exhibit model preference. SIPSO shows similar performance to AntEpiSeeker but with greater stability. However, SIPSO struggles to adapt to DNMEs. In terms of *F*-measure, ACOCMPMI significantly outperforms most compared methods in DMEs, though its performance is inferior to MACOED in DNMEs.

**Figure 4:**
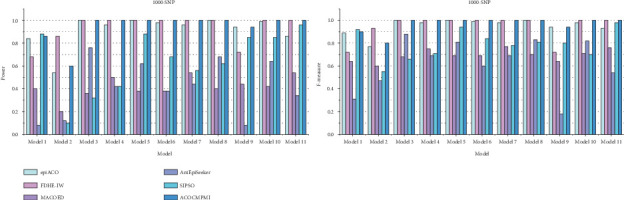
Detection power and *F*-measure of compared methods on large-scale datasets.

For large-scale datasets, detection power and F-measure of compared methods are presented in Figure 4. In terms of detection power, ACOCMPMI outperforms all compared methods in almost all datasets except Model 1–2 datasets. Performance of ACOCMPMI ranks second only to SIPSO in Model 1 datasets and to FDHE-IW in Model 2 datasets, respectively, further demonstrating the stability of its detection capability. AntEpiSeeker and MACOED show detection power ranging from 0.1 to 0.5 in most models, which is significantly lower than the detection power of ACOCMPMI. SIPSO performs effectively in datasets of Models 1, 5, and 9–11, but fails to identify over 60% of epistatic interactions in other datasets. epiACO and FDHE-IW exhibit detection power comparable to ACOCMPMI. epiACO performs well in most models since both it and ACOCMPMI use the ACO∗ and the information theory-based quantification measure. In terms of *F*-measure, ACOCMPMI has higher values than those of compared methods in almost all datasets except Model 1–2 datasets. Although epiACO and FDHE-IW are as effective as ACOCMPMI in identifying epistatic interactions in most models, their *F*-measure values vary widely among models, implying that both have weaker stability than ACOCMPMI. SIPSO, AntEpiSeeker, and MACOED generally have low *F*-measure values in most models, which is consistent with their performance in detection power.

**Figure 5:**
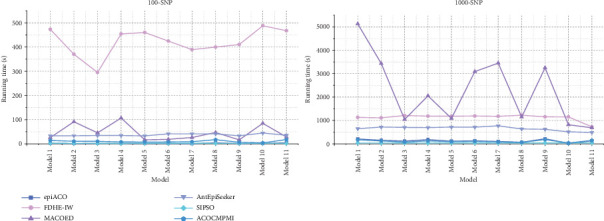
Running times of compared methods.

Running times of compared methods in different datasets are shown in Figure 5. It is seen that in small-scale datasets, ACOCMPMI has similar running times to those of both epiACO and SIPSO in various models. Running times of AntEpiSeeker in all models are relatively stable, though it takes more time than ACOCMPMI, epiACO, and SIPSO. MACOED shows significantly varying running times across models, implying that it is sensitive to model type. FDHE-IW requires unacceptable running times in all models. For large-scale datasets, in DMEs and DNMEs, ACOCMPMI has a clear advantage in terms of running time. Unlike FDHE-IW, which has the worst running times in small-scale datasets, MACOED becomes the most time-consuming method in large-scale datasets. Though SIPSO and epiACO have acceptable running times, their detection power is low.

**Figure 6:**
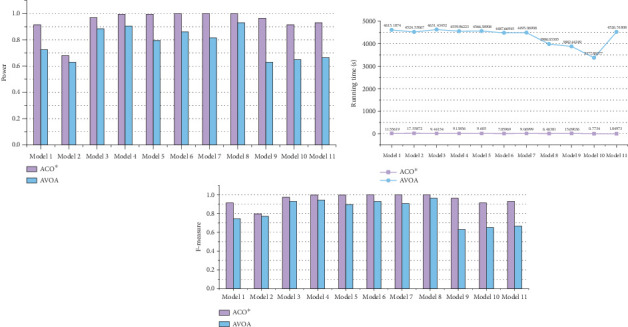
Detection power, *F*-measure, running time of ACO∗, and AVOA in small-scale datasets.

To demonstrate that the improved ACO∗ in ACOCMPMI is effective for searching epistatic interactions, ACO∗ is compared with AVOA in small-scale datasets, using CMPMI as their fitness function, in terms of detection power, *F*-measure, and running time, as shown in Figure 6. It is seen that even when facing the recently developed meta-heuristic algorithm AVOA, ACO∗ still has an advantage in search performance. In general, the random strategy and memory-filter strategy incorporated into the basic ACO∗ improve its detection capability without increasing running time.

### 4.4. Case Study

ACOCMPMI is applied to a real AMD dataset to detect two-order epistatic interactions. The AMD dataset contains 103,611 SNPs with 50 controls and 96 cases and has become a widely used benchmark dataset [39, 53]. ACOCMPMI runs four times on this AMD dataset, using ants and iterations as (10,000, 500), (10,000, 1000), (20,000, 250), and (20,000, 1000), respectively, to capture more epistatic interactions.  Table 2 lists the Top 15 detected epistatic interactions associated with AMD.

rs380390 is a G/A/T/C single-nucleotide variation in the CFH gene on human chromosome 1, and rs2019727, also located in CFH, is considered to be significantly associated with AMD in several studies [56–61]. rs3775652 is a C/T single-nucleotide variation located in the INPP4B gene on chromosome 4, and rs725518 is an A/G single-nucleotide variation in the RRM1 gene on chromosome 11, both of which have been detected as AMD-related SNPs [62, 63]. rs4772270 is a G/A/T/C single-nucleotide variation in the PCCA gene on chromosome 13, which has also been reported to be associated with AMD [55, 62, 63]. More recently, rs7863587 was reported to be highly associated with AMD [64]. Although further experiments and clinical studies are needed to confirm real epistatic interactions with AMD, we hope that these findings of ACOCMPMI can provide some clues for the pathological study of AMD.

## 5. Conclusions and Future Works

Epistatic interaction detection plays a pivotal role in understanding the genetic mechanisms underlying complex diseases. The effectiveness of epistatic interaction detection methods primarily depends on their interaction quantification measures and search strategies. Therefore, both are significant challenges for epistatic interaction detection. In this study, ACOCMPMI, a two-stage ACO∗ based on composite MPMI is proposed for detecting epistatic interactions. In the first stage, CMPMI is introduced to quantify epistatic interactions, and an improved ACO∗, incorporating filter and memory strategies, is employed to search for epistatic interactions. In the second stage, an exhaustive strategy and a BN score, that is, K2 score, are adopted to further identify epistatic interactions within the candidate SNP set obtained from the first stage. ACOCMPMI is compared with five state-of-the-art methods, including epiACO, FDHE-IW, AntEpiSeeker, SIPSO, and MACOED, using simulation data based on 11 epistatic interaction models. Furthermore, ACOCMPMI is applied to detect epistatic interactions in a real dataset related to AMD. The experimental results show that ACOCMPMI is an alternative method for epistatic interaction detection. The time complexity of ACOCMPMI is *O*(NT + nm^2^), where *N*, *T*, *n*, and *m* are numbers of ants, iterations, SNPs, and samples, respectively.

However, there are still several limitations in ACOCMPMI, which inspire us to continue working. First, how to adjust parameter settings to adapt to different scales of input SNP datasets should be further discussed. Second, the practical applicability and scalability of ACOCMPMI require a more detailed analysis. Although some of the identified SNPs have been validated, it remains unclear whether their two-order combinations are indeed causal factors of AMD. Furthermore, the current version of ACOCMPMI focuses on capturing two-order epistatic interactions. In reality, complex diseases are often caused by epistatic interactions with different orders, especially higher orders. Therefore, its future version should be developed to detect higher order epistatic interactions.

## Figures and Tables

**Table 1:** Details of epistatic interaction models.

**Models**	**MAF(*a*)**	**MAF(*b*)**	***AABB***	***AABb***	***AAbb***	***AaBB***	***AaBb***	***Aabb***	***aaBB***	***aaBb***	***aabb***
Model 1	0.2	0.2	0.087	0.087	0.087	0.087	0.146	0.190	0.087	0.190	0.247
Model 2	0.5	0.5	0.009	0.009	0.009	0.013	0.006	0.006	0.013	0.006	0.006
Model 3	0.5	0.5	0.092	0.092	0.092	0.092	0.319	0.319	0.092	0.319	0.319
Model 4	0.2	0.2	0.084	0.084	0.084	0.084	0.210	0.210	0.084	0.210	0.210
Model 5	0.5	0.5	0.052	0.052	0.052	0.052	0.137	0.137	0.052	0.137	0.137
Model 6	0.5	0.5	0.072	0.164	0.164	0.164	0.072	0.072	0.164	0.072	0.072
Model 7	0.5	0.5	0.067	0.155	0.155	0.155	0.067	0.067	0.155	0.067	0.067
Model 8	0.3	0.3	0.486	0.960	0.538	0.947	0.004	0.811	0.640	0.606	0.909
Model 9	0.2	0.5	0.103	0.063	0.124	0.098	0.086	0.069	0.021	0.147	0.059
Model 10	0.5	0.5	0.000	0.000	0.000	0.000	0.050	0.000	0.100	0.000	0.000
Model 11	0.3	0.3	0.000	0.020	0.000	0.020	0.000	0.020	0.000	0.020	0.000

**Table 2:** Top 15 detected epistatic interactions associated with AMD.

**SNP 1**			**SNP 2**			**Fitness value**	**p** ** value**	**Times**
**Name**	**Gene**	**Chr**	**Name**	**Gene**	**Chr**			
rs3775652	INPP4B	4	rs380390	CFH	1	123.48	0.0175	3
rs3775652	INPP4B	4	rs725518	RRM1	11	121.99	0.0149	3
rs380390	CFH	1	rs725518	RRM1	11	121.05	0.0039	3
rs380390	CFH	1	rs54816	RRM1	11	120.19	0.0064	3
rs3775652	INPP4B	4	rs54816	RRM1	11	119.90	0.0081	3
rs7863587	/	9	rs380390	CFH	1	119.63	0.0415	2
rs4772270	PCCA	13	rs380390	CFH	1	118.82	0.0265	1
rs6480996	/	10	rs380390	CFH	1	118.11	0.0052	1
rs2019727	CFH	1	rs380390	CFH	1	118.05	0.0082	1
rs380390	CFH	1	rs365299	/	1	117.10	0.0190	1
rs3775652	INPP4B	4	rs4772270	PCCA	13	115.98	0.0458	1
rs7863587	/	9	rs3775652	INPP4B	4	115.49	0.0394	1
rs3775652	INPP4B	4	rs3775650	INPP4B	4	113.31	0.0234	1
rs3775650	INPP4B	4	rs4772270	PCCA	13	112.69	0.0046	1
rs7863587	/	9	rs725518	RRM1	11	111.21	0.0285	1

## Data Availability

Data is available on request.
